# DELAY OF GERMINATION 1, the Master Regulator of Seed Dormancy, Integrates the Regulatory Network of Phytohormones at the Transcriptional Level to Control Seed Dormancy

**DOI:** 10.3390/cimb44120423

**Published:** 2022-12-08

**Authors:** Qiujia Li, Xi Chen, Shengnan Zhang, Siyao Shan, Yong Xiang

**Affiliations:** 1 Shenzhen Branch, Guangdong Laboratory for Lingnan Modern Agriculture, Genome Analysis Laboratory of the Ministry of Agriculture, Agricultural Genomics Institute at Shenzhen, Chinese Academy of Agricultural Sciences, Shenzhen 518120, China; 2 Center for Crop Science, College of Agronomy, Qingdao Agricultural University, Qingdao 266109, China

**Keywords:** dormancy, DOG1, phytohormone, ABA, GA, IAA

## Abstract

Seed dormancy, an important adaptive trait that governs germination timing, is endogenously controlled by phytohormones and genetic factors. DELAY OF GERMINATION 1 (DOG1) is the vital genetic regulator of dormancy, significantly affecting the expression of numerous ABA and GA metabolic genes. However, whether DOG1 could influence the expression of other phytohormone-related genes is still unknown. Here, we comprehensively investigated all well-documented hormone-related genes which might be affected in *dog1–2* dry or imbibed seeds by using whole-transcriptome sequencing (RNA-seq). We found that DOG1 could systematically control the expression of phytohormone-related genes. An evident decrease was observed in the endogenous signal intensity of abscisic acid (ABA) and indole-3-acetic acid (IAA), while a dramatic increase appeared in that of gibberellins (GA), brassinosteroids (BR), and cytokinin (CK) in the *dog1–2* background, which may contribute considerably to its dormancy-deficient phenotype. Collectively, our data highlight the role of DOG1 in balancing the expression of phytohormone-related genes and provide inspirational evidence that DOG1 may integrate the phytohormones network to control seed dormancy.

## 1. Introduction

Seed dormancy, defined as the incapacity of a viable seed to germinate under favorable conditions [1], is a critical adaptive trait that determines the seed germination timing and facilitates seedling survival. Seed dormancy is also an invaluable agronomic trait that controls fast and uniform germination, especially preventing pre-harvest sprouting which causes tremendous economic losses [2,3,4].

Seed dormancy is tightly controlled by phytohormones and genetic factors. Nine major phytohormones have been discovered and well studied to date, which are abscisic acid (ABA), auxin (the major natural auxin in plants is indole-3-acetic acid, IAA), brassinosteroids (BR), cytokinins (CK), ethylene (ET), gibberellins (GA), jasmonic acid (JA), salicylic acid (SA), and strigolactones (SL) [5,6,7]. ABA and GA are undoubtedly the two principal hormones that regulate seed dormancy, and the balance between GA and ABA is critical for regulating seed dormancy and germination [8,9]. ABA positively regulates dormancy [1,6]. During seed maturation, ABA levels gradually increase to establish seed dormancy; while during seed germination, ABA is gradually inactivated to release dormancy [10]. Generally, mutants with increased ABA contents or enhanced ABA signaling exhibit elevated dormancy levels, while those with decreased ABA contents or attenuated ABA signaling display reduced dormancy levels [1,2,3,4,5,6]. GA, which acts antagonistically to ABA, breaks dormancy and promotes germination. Like ABA, mutants with altered GA contents or GA signaling display varied dormancy levels [1,2,3,4,5,6]. Some phytohormones regulate seed dormancy via the interaction with ABA and GA. ET, another key phytohormone besides ABA and GA, negatively regulates seed dormancy by counteracting ABA effects through the regulation of ABA metabolism and signaling pathway [11,12,13]. IAA, a newly recognized positive regulator of seed dormancy after ABA, can maintain seed dormancy through the stimulation of ABI3-mediated ABA signaling [14]. BR and CK can stimulate seed germination via the interaction with GA [15,16]. Despite few reports showing the role of JA, SL, and SA in regulating seed dormancy, they can regulate seed germination. JA inhibits seed germination synergistically with ABA [17], and SL can stimulate seed germination circumventing the GA requirement [18], SA inhibits seed germination under normal conditions while improving it under salt stress conditions [19].

DELAY OF GERMINATION 1 (DOG1) is the major genetic regulator of seed dormancy. DOG1 is closely associated with the degree of dormancy in freshly harvested seeds [20,21,22]. So many genes modulate seed dormancy dependent on their regulation of DOG1 expression from various aspects, ranging from the *cis*-element within the DOG1 promoter to the chromatin remodeling at the DOG1 locus, such as Basic LEUCINE ZIPPER TRANSCRIPTION FACTOR 67 (bZIP67), ETHYLENE RESPONSE FACTOR 12 (ERF12), the transcript elongation factor TFIIS, and two histone demethylases, LYSINESPECIFIC DEMETHYLASE LIKE 1 and 2 (LDL1 and LDL2) [23,24,25,26]. Despite numerous reports on upstream regulators of DOG1, there are few reports on downstream targets of DOG1. DOG1 regulates seed germination and flowering time through an influence on levels of microRNAs (miRNAs) miR156 and miR172 [27]. Furthermore, DOG1 physically interacts with two key negative regulators of ABA signaling ABA HYPERSENSITIVE GERMINATION1 (AHG1) and AHG3 to functionally block their essential downstream roles [28,29], implying a somewhat convergence of the DOG1 pathway with ABA signaling pathway. In *dog1–1*, a loss-of-function of DOG1 mutant, the expression of some ABA synthesis genes (*NCED2/3/5/9*) [30,31,32] was significantly reduced, while the expression of ABA catabolism genes (*CYP707A1/A3*) [33,34] and GA synthesis (*GA20ox1/2/3* and *GA3OX1/2*) [35,36] was significantly increased; ABA and GA contents also changed in *dog1–1* mutants corresponding to the gene expression [20]. These clues suggest an important role of DOG1 in modulating the expression of ABA and GA metabolism-related genes, which may contribute considerably to the regulation of seed dormancy by DOG1. Apart from ABA and gibberellins, more and more evidence indicates that other phytohormones also function in seed dormancy individually or by hormones crosstalk. As a seed dormancy core regulator, DOG1 may also affect these phytohormones’ metabolism and signaling. However, due to the technical limitations at that time, the relationship between the function of DOG1 and the expression regulation of phytohormone-related genes could not be investigated in a high-throughput way. In this study, we used RNA-seq to systematically analyze the impact of DOG1 on the expression of all known important hormone-related genes and found that the influence is tremendous but distinct for a particular phytohormone. Moreover, DOG1 negatively regulates the dormancy hormone represented by GA and positively regulates the dormancy hormone represented by ABA and IAA, and their balance is important for establishing and maintaining seed dormancy. As a whole, our work reconfirms the role of DOG1 in regulating hormone-related gene expression, and provides an inspirational clue that DOG1 may function as a master regulator of nine dormancy-related phytohormones.

## 2. Materials and Methods

### 2.1. Plant Materials and Growth Condition

Arabidopsis seeds used in this study were Col-0 (wild type) and the *dog1–2* mutant, which is in the Col-0 background [20]. Seeds were sown in the soil mixture (potting soil: vermiculite = 3:1) and grown in the growth chamber with 16-h-light under 80–90 μmol m^−2^ s^−1^ white light intensity/8-h-dark cycle at 24 °C/22 °C.

### 2.2. Germination Assay

Seed germination assays were performed as previously described [20]. Freshly harvested seeds of each genotype were sown on filter paper, placed into transparent moisturized containers, and incubated in a germination cabinet in long-day conditions. Photos that check the germination status of Col and *dog1–2* after imbibition for 6 h were taken under a stereomicroscope (Olympus, Tokyo, Japan); photos that check the surface structure of Col and *dog1–2* seeds after imbibition for 6 h were taken using a scanning electron microscope (Hitachi, Tokyo, Japan). The germination percentages were analyzed after 7 days of incubation. For each germination test, we performed ten biological replicates.

### 2.3. Transcriptome Analysis

Freshly harvested seeds were immediately sampled (dry seeds) or imbibed in saturated filter paper for 6 h prior to sample (imbibed seeds). RNA was extracted from dry and imbibed seeds using FastPure Plant Total RNA Isolation Kit (Polysaccharides and Polyphenolics-rich) (Vazyme Biotech, Nanjing, China) according to the manufacturer’s instructions. RNA-Seq library preparation and sequencing were performed at Novogene Biotech (Beijing, China) using an Illumina Novaseq500-PE150. Low-quality sequences were removed using fastp (version 0.20.0, -z 4 -q 20 -u 30 -n 10). We then mapped the clean reads to the Arabidopsis reference genome using the software Hisat2 (version 2.1.0). FeatureCounts (version 2.0.1) was used to count the reads using these common parameters (-*p* -t exon -g gene_id). Raw counts were normalized with DEseq2 (version 1.34.0) to compare gene expression in different samples. Gene expression values (normalized counts) were calculated by DEseq2. DEGs were also identified with DEseq2 with adjust *p* < 0.05 and fold-changes > 2.

### 2.4. RT-qPCR Assay

Total RNA was extracted using FastPure Plant Total RNA Isolation Kit (Polysaccharides and Polyphenolics-rich) (Vazyme Biotech, Nanjing, China) according to the manufacturer’s instructions. After treatment with DNase I to remove residual genomic DNA, 1 μg total RNA was used as template for reverse transcription reaction using HiScript^®^ III 1st Strand cDNA Synthesis Kit (Vazyme Biotech, Nanjing, China). Quantitative real-time PCR was performed using ChamQ Universal SYBR qPCR Master Mix (Vazyme Biotech, Nanjing, China). Relative gene expression was analyzed based on the 2^−ΔΔCt^ method with *ACTIN8* (AT1G49240) as an internal control. Gene-specific primers are list in Table S3.

## 3. Results

### 3.1. Dog1–2 Is Suitable to Analyze DOG1 Regulatory Role

**Figure 1:**
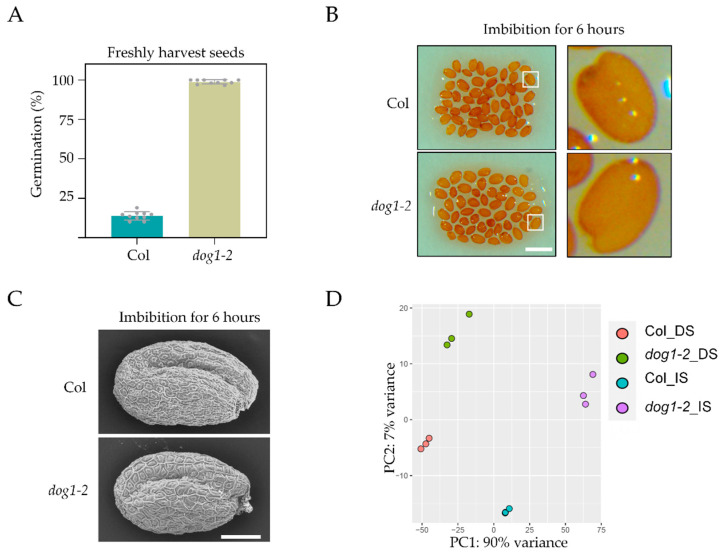
Quality evaluation of the experimental materials, conditions, and data. (**A**) The germination rate showed that the *dog1–2* seeds completely lost dormancy. (**B**) The stereomicroscope photos showed that the germination status of the *dog1–2* seeds was not changed compared with that of Col after imbibition for 6 h. (**C**) The scanning electron microscopy images showed that the surface structure of *dog1–2* seeds was not changed compared with that of Col after imbibition for 6 h. (**D**) Two-dimensional Principal Component Analysis (PCA) showed that the whole transcriptome level of the *dog1–2* seeds was significantly altered compared with that of the wild-type. Scale bars, 1 mm in B and 120 μm in C.

We adopted Col and *dog1–2* seeds with clearer genetic backgrounds as materials to investigate the transcriptome changes in this study. *dog1–2* has been verified as a loss of function mutant in the Col-0 background and showed no dormancy [20,37]. We also confirmed the reduced dormancy phenotype before the following treatment (Figure 1A). Imbibition is the early stage of seed germination in which the transcriptome changed dramatically. In order to capture the DOG1-regulated transcription changes, the time point of sampling imbibed seeds should rule out the disruption of the change in seed germination status or seed surface structure. Therefore, we assessed the seeds structure of Col and *dog1–2* with the help of the stereomicroscope and the scanning electron microscope at the imbibition stage. No obvious difference could be observed between Col and *dog1–2* when the seeds were imbibed for 6 h (Figure 1B,C), which implied that 6-h imbibition was suitable for transcription analysis. Therefore, seeds were sampled at 0 (hereafter referred to as dry seeds) and 6 h (hereafter referred to as imbibed seeds) after the start of imbibition. Finally, we performed an RNA-seq experiment with three biological replicates and comparatively analyzed the gene expression differences between wild-type Col and the *dog1–2* mutant. Two-dimensional Principal Component Analysis (PCA) showed that transcriptomes of *dog1–2* were different from Col in both dry and imbibed seeds (Figure 1D). In addition, the three biological repeats were always clustered together, which indicated the high reproducibility of this dataset. Taken together, the RNA-seq data obtained from the dry and imbibed seeds of Col and *dog1–2* mutant is reliable to investigate the influence of DOG1 on the whole transcriptome level.

### 3.2. DOG1 Dramatically Influences the Whole Transcriptome Level of Freshly Harvested Seeds

**Figure 2:**
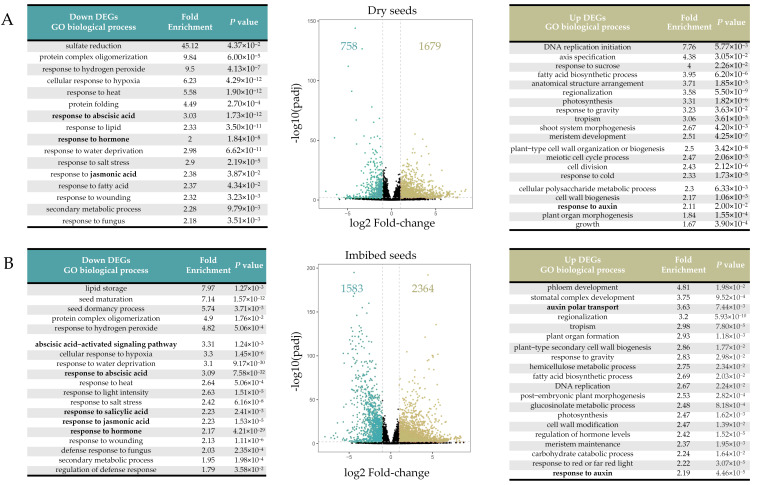
The overview of the alteration of transcriptome level in *dog1–2* seeds. The volcano plots (middle panel) and the tables (left and right panels) showed the number and extent of up-and down-regulated DEGs and the Top categories of GO analysis in dry (**A**) and imbibed seeds (**B**).

Our RNA-seq method detected a total of 25280 expressed genes in freshly-harvested dry and imbibed seeds. The genes with absolute fold-change > 2 and *p*-value < 0.05 were defined as differentially expressed genes (DEGs). The up-regulated and down-regulated DEGs were 1679 and 758 in dry seeds, respectively (Figure 2A and Table S1). The up-regulated and down-regulated DEGs were 2364 and 1583 in imbibed seeds, respectively (Figure 2B and Table S1). The Gene ontology (GO) enrichment analysis summarized the top categories in which the DOG1-regulated DEGs were involved. As expected, we noticed a considerable number of categories were associated with phytohormones in dry and imbibed seeds. Notably, ABA-related categories (GO: 0009737/0009738) were enriched highest among all phytohormones, which was 3.03- and 3.31-fold enrichment in dry and imbibed seeds, respectively. The category of “response to auxin” (GO: 0009733) was enriched 2.11- and 2.19-fold significantly in the up-regulated DEGs both in dry and imbibed seeds, respectively, suggesting a link between DOG1 and gene expression of IAA. Unexpectedly, some phytohormones, such as salicylic acid and jasmonic acid, which are generally thought to play weak roles in regulating seed dormancy, were also included in the top categories (Figure 2), implying that DOG1 is more closely related to phytohormones than previously thought.

### 3.3. DOG1 Plays an Important Role in Regulating Phytohormone-Related Gene Expression

**Figure 3:**
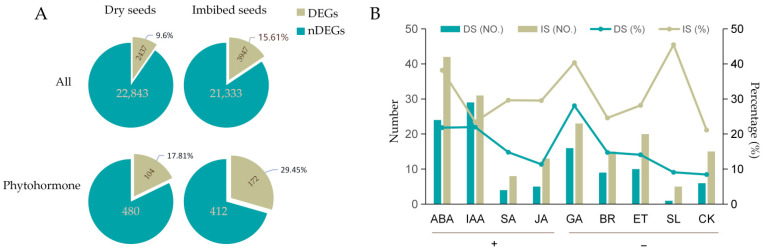
Phytohormone-related DEGs were highly enriched in *dog1–2*. (**A**) The percentage of the total DEGs and the phytohormone-related DEGs in dry and imbibed seeds. DEGs, differentially expressed genes. nDEG, not DEGs. (**B**) The number and percentage of DEGs in the indicated phytohormone pathways in dry and imbibed seeds. “+” denotes the phytohormones that induce seed dormancy or inhibit seed germination; “−” denotes the phytohormones that release seed dormancy or promote seed germination. DS, dry seeds; IS, imbibed seeds.

The complicated gene regulatory networks of phytohormone have been studied intensively over the past decades. We carefully selected 636 genes proven to be involved in the homeostasis or signal transduction of phytohormones (Table S2) from the classic publications for gene expression detection in *dog1–2* background. Among them, the expression value of 585 genes was detected by our RNA-seq. The total DEGs were approaching and even exceeding one-tenth of the total detectable genes in dry seeds (2437 DEGs, 9.6%) and imbibed seeds (3947 DEGs, 15.61%) in the *dog1–2* mutant, respectively (Figure 3A). When considering the phytohormone-related DEGs only, these proportions climbed up to one-fifth (104 DEGs, 17.81%) and one-third (172 DEGs, 29.45%) in dry and imbibed seeds, respectively. The enrichment degree of hormone-related DEGs in dry and imbibed seeds was almost twice that of the whole DEGs (17.81% vs. 9.6%; 29.45% vs. 15.61%) (Figure 3A), indicating that the functional DOG1 is especially important for the normal expression of phytohormones related genes.

We further analyzed the specific effect of DOG1 on a single hormone. We divided plant hormones into two groups according to their roles in seed dormancy or seed germination. One group, denoted by “+”, includes the hormones that can induce seed dormancy or inhibit seed germination; the other group, denoted by “−”, includes those that can release seed dormancy or promote seed germination. In dry seeds, the order of the percentage of DEGs in each hormone from high to low is ABA, IAA, SA, and JA for group “+” and GA, BR, ET, SL, and CK for group “−”; in imbibed seeds, the order is ABA, SA, JA, and IAA for group “+” and SL, GA, ET, BR, and CK for group “−” (Figure 3B). ABA and GA are ranked among the top both in dry seeds and imbibed seeds, indicating that DOG1 had the greatest influence on ABA- and GA-related gene expression. The number and the percentage of DEGs of most hormones increased significantly after seed imbibition (with the only exception of IAA), suggesting that the function of DOG1, the master regulator of seed dormancy, in regulating gene expression in the early stage of seed germination should be considered.

### 3.4. DOG1 Differentially Affects the Homeostasis or Signaling DEGs of Different Hormones

**Figure 4:**
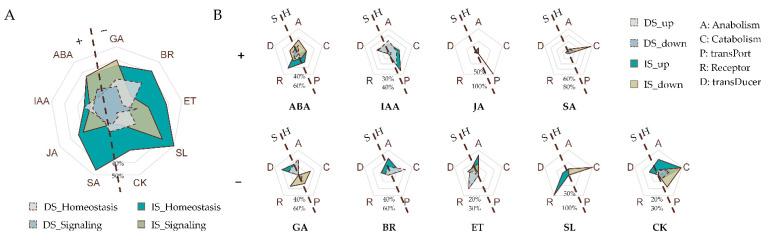
The homeostasis or signaling DEGs of different phytohormones were variously affected in *dog1–2*. (**A**) The proportion of the homeostasis and signaling DEGs of phytohormones. The dashed line divides the phytohormones into two classes: “+” denotes the phytohormones that induce seed dormancy or inhibit seed germination; “−” denotes the phytohormones that release seed dormancy or promote seed germination. DS, dry seeds; IS, imbibed seeds. (**B**) The proportion of up- or down-regulated DEGs involved in the anabolism, catabolism, and transport, as well as the signal reception and transduction of the indicated phytohormone. The dashed line divides the five categories into two classes: S, Signaling; H, Homeostasis.

Both homeostasis and signaling are necessary for hormone proper function. Therefore, we compared the effects of DOG1 on the expression of different phytohormone homeostasis or signaling genes. The percentage of homeostasis-related DEGs is usually higher than that of signaling-related DEGs both in dry seeds and in imbibed seeds for most hormones (Figure 4A), indicating that DOG1 had a more notable impact on phytohormone homeostasis. However, there is an obvious exception for ABA, in which the percentage of homeostasis-related DEGs is lower than that of signaling in dry seeds and is about the same amount in imbibed seeds, suggesting that the regulation of DOG1 on ABA is somehow different from other hormones.

Of a particular hormone within a plant cell, the homeostasis is regulated both by the rate of its anabolism and catabolism and by the capacity of its transport; its signaling is regulated both by the receptors (responsible for signal perception) and by the transducers (responsible for signal transduction). Therefore, we compared the impacts of DOG1 on the expression of genes related to the above five aspects of each hormone. DOG1 showed completely distinct regulatory patterns for different hormones. For hormones such as ABA, IAA, and GA, which play a predominant role in the regulation of seed dormancy, DOG1 had a relatively balanced regulation of the expression of the genes involved in the above five aspects (Figure 4B). However, for hormones such as JA, SA, and SL, which play weak roles in dormancy regulation, the regulation pattern of gene expression by DOG1 is quite irregular (Figure 4B).

### 3.5. DOG1 Has a Specific Impact on the Expression of a Particular Gene in Various Phytohormones

**Figure 5:**
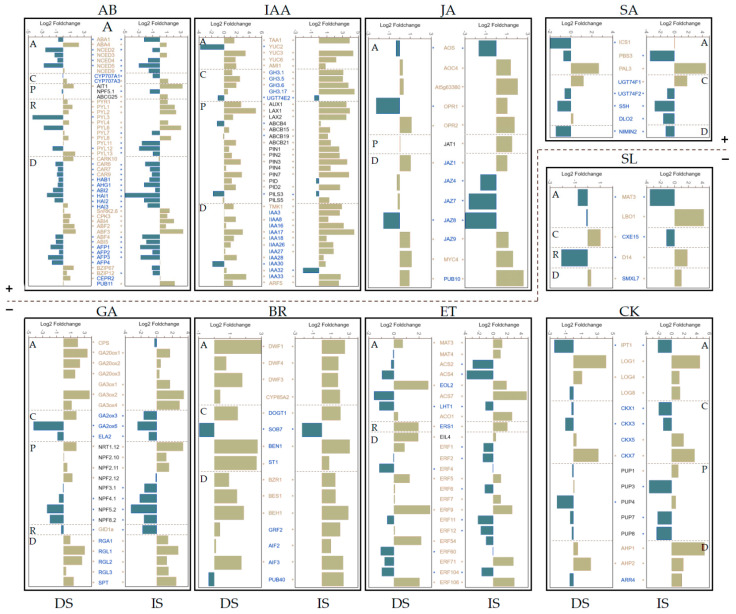
Comprehensive analysis of the influence of DOG1 on the expression of a particular gene in each phytohormone. The histogram shows expression alteration of the up- or down-regulated DEGs in *dog1–2* in the indicated phytohormone pathway. Gene name in yellow or blue denotes that the gene plays a positive or negative role in homeostasis or signal transduction, respectively. *, *p* < 0.05. The dashed line divides the phytohormones into two classes: “+” denotes the phytohormones that induce seed dormancy or inhibit seed germination; “−” denotes the phytohormones that release seed dormancy or promote seed germination. DS, dry seeds; IS, imbibed seeds. A, Anabolism; C, Catabolism; P, transPort; R, Receptor; D, transDucer.

The completely distinct regulatory pattern of DOG1 on the genes in different hormones prompted us to dissect the specific influence of DOG1 on the expression of a particular gene in each hormone (Figure 5 and Figure S1). For GA, we observed a consistent phenomenon, that is all the GA anabolism-related DEGs (*CPS*, *GA20ox1/2/3*, and *GA3ox1/2/4*) were up-regulated while all the GA catabolism-related DEGs (*GA2ox3/6* and *ELA2*) were down-regulated in *dog1–2* seeds (Figure 5). For ABA, we also observed a similar phenomenon, but with two exceptions (*ABA4* and *CYP707A1*); that is, most ABA anabolism-related DEGs (*ABA1* and *NCED2/3/4/5/9*) were down-regulated while one ABA catabolism-related DEG (*CYP707A3*) was up-regulated in *dog1–2* seeds. These results emphasized the importance of DOG1 in regulating ABA and GA homeostasis. The impact of DOG1 on ABA signaling is vast, too. DOG1 could significantly affect the expression of genes encoding almost all the core components in ABA signaling, including the majority of indispensable ABA receptors (*PYR1/PYL1/2/3/4/6/7/8/11/12/13*), the majority of pivotal negative regulators PP2Cs (*ABI2*, *AHG1*, *HAB1*, and *HAI1/2/3*), several critical down-stream kinases (*SnRK2.6* and *CPK3*), numerous crucial transcription factors (*ABI4*, *ABI5*, and *ABFs*), and multiple vital regulatory factors for these core-components such as *CARK*, *CARs*, and *CEPR2* (for ABA receptors), as well as *PUB11* (for PP2Cs) and *AFP1–4* (for ABI5). This phenomenon highlights the importance of DOG1 in ABA signaling, consistent with the recent discovery that DOG1 can directly interact with phosphatases AHG1 in ABA signaling, and block their downstream roles [28,29].

DOG1 also had an immense impact on the gene expression of GA-signaling. The expression levels of three GA receptors decreased in *dog1–2* to varying degrees, especially the *GID1a* (Figure 5); nearly all the expression of genes encoding GA signal transducer increased in *dog1–2*, including four of five pivotal DELLAs (*RGA1*, *RGL1*, *RGL2*, and *RGL3*) (Figure 5), suggesting a role of DOG1 in GA signaling. DOG1 also affected the expression of other hormone-related genes to varying degrees. For example, most anabolism-related genes of IAA, JA, BR, and CK were up-regulated in *dog1–2* seeds; however, most catabolism-related genes in IAA and BR were also up-regulated. Most transport-related genes of IAA and JA were up-regulated in *dog1–2* seeds, while those of CK were down-regulated (Figure 5). Generally, the impact of DOG1 on gene expression for a particular hormone includes positive and negative; however, it is worth noting that the majority of IAA- and BR-related DEGs were only up-regulated in *dog1–2* seeds, implying that DOG1 has a special impact on these two hormones. In a nutshell, for ABA and GA, the two hormones that play dominant roles in regulating seed dormancy, the expression profile affected by DOG1 is obvious and regular; however, for other hormones which may play minor roles in regulating seed dormancy, there was no obvious regular pattern by DOG1.

### 3.6. DOG1 Ultimately Affects the Endogenous Signal Intensity of Various Hormones

**Figure 6:**
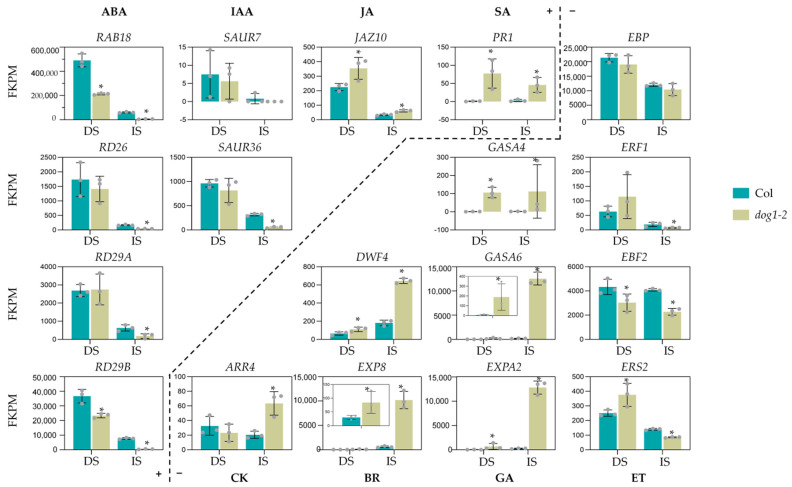
DOG1 differentially regulates the endogenous signal intensity of different phytohormones. FKPM value shows the relative expression level of representative marker genes of the indicated phytohormone pathway in Col and *dog1–2* seeds. DS, dry seeds; IS, imbibed seeds. The dashed line divides the phytohormones into two classes: “+” denotes the phytohormones that induce seed dormancy or inhibit seed germination; “−” denotes the phytohormones that release seed dormancy or promote seed germination. *, *p* < 0.05.

Considering that DOG1 could regulate the expression of various hormone-related genes, we compared the expression alteration of marker genes of each hormone between WT and *dog1–2* mutants to evaluate the final impact of DOG1 on the signal intensity of a particular hormone. The expression alterations of marker genes of a given hormone are widely adopted to reflect the changes in signal intensity due to the variation of contents or sensitivity of that hormone. We selected two to four widely accepted reporter genes from the literature for each hormone: *RAB18*, *RD26*, *RD29A*, and *RD29B* for ABA [38,39,40]; *SAUR7*, *SAUR36*, and *SAUR46* for IAA [41,42]; *JAZ10*, *JR2*, and *VSP2* for JA [43,44]; *PR1*, *PR2*, and *PR5* for SA [45]; *GASA4*, *GASA6* and *EXPA2* for GA [46,47,48]; *EBP*, *ERF1*, *EBF2* and *ETR2* for ET [49,50,51]; *DWF4*, *EXP8*, and *SAUR-AC1* for BR [52,53]; and *ARR4* and *ARR5* for CK [46]. The evaluation of SL is missed because there is currently no widely recognized and used marker gene for SL. Some marker genes may not be suitable as indicators of hormonal levels within seeds because their expression levels are hardly detected in this tissue (Figure S2), although they may work well in other tissues such as roots or rosettes. After excluding the interference of these marker genes, we obtained perspicuous results (Figure 6). The expression of all marker genes of ABA decreased significantly in *dog1–2*, while that of GA increased sharply, reconfirming that DOG1 can disrupt the balance between endogenous ABA and GA. The signal intensity of IAA, another essential hormone that induces seed dormancy besides ABA, also decreased in imbibed seeds reflected by its marker gene *SAUR36*. The signal intensity of JA and SA, two hormones that may play weak roles in seed dormancy but mainly function in repressing seed germination, increased both in dry seeds and imbibed seeds. Like GA, the signal intensity of CK, and BR, two hormones that negatively regulate seed dormancy, both increased to some extent. The only hormone that was difficult to determine the alteration of signal intensity was ET. Among the four marker genes of ET, one did not change (*EBP*), two changes were contradictory in dry and imbibed seeds (*ERF1* and *ERS2*), and one decreased, but the degree was very slight.

In addition, the expression pattern of marker genes was further analyzed by RT-qPCR (Figure S3). The expression of most marker genes was significantly altered in *dog1–2*. ABA-related marker genes decreased significantly in *dog1–2,* while all marker genes of CK and GA increased. As the two marker genes of IAA, *SAUR7* and *SAUR36* showed slight decrease in dry and imbibition seeds, respectively. The RT-qPCR results were highly consistent with our RNA-seq data.

## 4. Discussion

As the core regulator of seed dormancy, DOG1 was frequently reported as a downstream factor that can be regulated directly and indirectly [23,24,25,26], while few reports about the downstream targets of DOG1 [27,28,29]. Previous study has reported the severely affected transcriptome in *dog1–1* with microarray analysis [54]. However, the *dog1–1* mutant is in a complicated NIL-DOG1 background, a near-isogenic line containing the DOG1 allele from Cvi in a Ler background. In this study, we performed a transcriptome analysis using dry and imbibed seeds of *dog1–2* in Col-0 background with RNA-seq analysis. The time point of samples collection was precisely selected to detect whole transcriptome at the beginning of seed germination according to the seeds` morphological status (Figure 1). The PCA analysis revealed the high reproducibility of our dataset. Generally, our study could present a more reliable investigation into the influence of DOG1 on the whole transcriptome.

In our data, DOG1 influences gene expression significantly because one-tenth of genes, especially hormone-related genes, were differentially expressed in *dog1–2* dry and imbibed seeds (Figure 2 and Figure 3A). Hormone-related genes, with their roles quickly in response to endogenies and exogenous cues, effected more remarkable in our study, as the proportion of DEGs reached nearly one-fifth and almost one-third in dry and imbibed seeds, respectively (Figure 3A). The effects of DOG1 on the expression of genes in a particular phytohormone are quite distinct (Figure 4 and Figure 5), and might lead to various degrees of changes in the signal intensity of each hormone (Figure 6). Those indicate analysis downstream is also quite important to uncover the DOG1 function.

Further investigation revealed an evident decrease in the endogenous signal intensity of ABA and IAA while a dramatic increase in that of GA, BR, and CK (Figure 5 and Figure 6). Our data obtained from Col and *dog1–2* are highly consistent with the previous results [20], and indicate that DOG1 may be at the center of hormonal regulation, especially reconfirming that DOG1 plays a central role in regulating the balance between GA and ABA. Unlike GA and ABA, the relationship between DOG1 and IAA has been overlooked for a long time until a recent work combined transcriptome and translatome analyses highlighted the role of auxin biosynthesis in the control of DOG1-dependent seed dormancy [55]. Bai et al. found that the expression of IAA metabolism genes was discriminative during the imbibition of after-ripened and dormant seeds of NIL-DOG1 [55]. This implied the role of IAA in dormancy and the possible link between IAA and DOG1. In this work, we also identified a large number of DEGs involved in the IAA pathway, including *YUC3* and *YUC6*, encoding two essential auxin synthesis enzymes [56,57], as well as other genes involved in the auxin pathway (Figure 5). Therefore, the relationship between DOG1 and IAA deserves in-depth studies in the future. Besides ABA, GA, and IAA, DOG1 can also influence the endogenous signal intensity of JA, SA, CK, and BR (Figure 5). There are few reports about those hormones involved in DOG1-related seed dormancy, which might be due to their minor effect or not clearly characterized roles in dormancy regulation.

DOG1 has such a substantial effect on gene expression, which might potentially be explained by its protein sequence. One of the three protein domains of DOG1 is present in group D bZIP transcription factors, and the protein with the highest sequence similarity to DOG1 is the wheat transcription factor HBP-1b [58]. DOG1 may regulate dormancy-related phytohormone genes potentially through acting as a DNA-binding protein. Another possibility is that DOG1 may interact with some transcription factors and coordinately regulate downstream gene expression. Nee and Nishimura identified many DOG1 interacting proteins by pull-down combined with mass spectrometry, including a large number of genes that may function as transcription factors [28,29]. The third possibility is the feedback from the DOG1-involved pathway. For example, DOG1 interacts with AHG1 and regulates the activation state of SnRK2s through the inhibition of the PP2C activity of AHG1 [28,29]. That might induce an ABA feedback response during the biological process.

To uncover these possibilities, further validation of DOG1 co-effectors, especially DOG1 binding to those DNA sites by employing other methods such as Chromatin immunoprecipitation combined with next-generation sequencing (ChIP-seq) is required.

## 5. Conclusions

Our study uncovered a dramatic transcriptome alteration in dry and imbibed seeds of *dog1–2,* a non-dormant mutant in Col-0 background. Further investigation revealed that phytohormone-related DEGs referred to both homeostasis and signaling pathway. The signal intensity of ABA and IAA decreased significantly, while that of GA, CK, and BR increased notably. Our data indicated the center-balancing role of DOG1 in phytohormones related dormancy control.

## Data Availability

All data in this study can be found in public databases and Supplementary Materials, as described in the Section 2.

## Supplementary Materials

The following supporting information can be downloaded at: https://www.mdpi.com/article/10.3390/cimb44120423/s1, Figure S1: Comprehensive analysis of the influence of DOG1 on the expression of a particular gene in each phytohormone. Figure S2: The expression value of marker genes that are not suitable as indicators of hormonal levels within seeds. Figure S3: Validation of the expression of marker genes by RT-qPCR assays. Table S1. All DEGs list in dry seeds and imbibition seeds. Table S2. The Hormone-related genes list. Table S3. Primers used for RT-qPCR.
