# Supplementary material for: DELAY OF GERMINATION 1, the Master Regulator of Seed Dormancy, Integrates the Regulatory Network of Phytohormones at the Transcriptional Level to Control Seed Dormancy
Source: Curr Issues Mol Biol. 2022 Dec 8;44(12):6205–17. doi: 10.3390/cimb44120423 (PMC9777134; doi:10.3390/cimb44120423)
Supplement: Supplementary file 1 [file cimb-44-00423-s001.zip › Supplementary Figures.pdf]

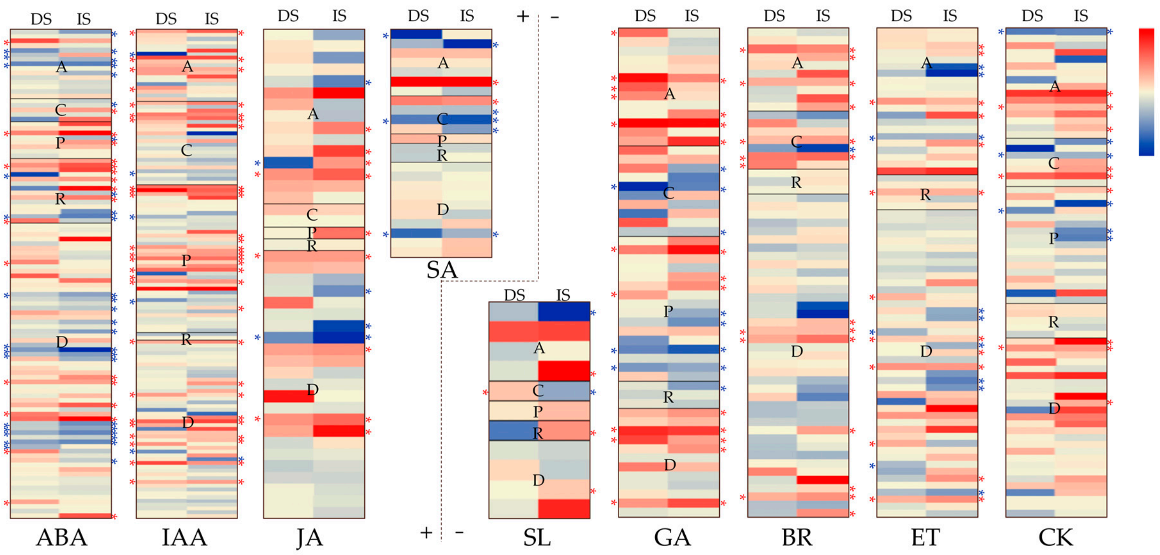

Figure S1. Comprehensive analysis of the influence of DOG1 on the expression of a particular gene in each phytohormone. The heatmap shows the expression alteration of the detectable phytohormone-related genes listed in Table S1. “*” indicates the DEGs. The dashed line divides the phytohormones into two classes: “+” denotes the phytohormones that induce seed dormancy or inhibit seed germination; “-” denotes the phytohormones that release seed dormancy or promote seed germination. DS, dry seeds; IS, imbibed seeds. A, Anabolism; C, Catabolism; P, transPort; R, Receptor; D, transDucer. Red, up-regulation; Blue, down-regulation.

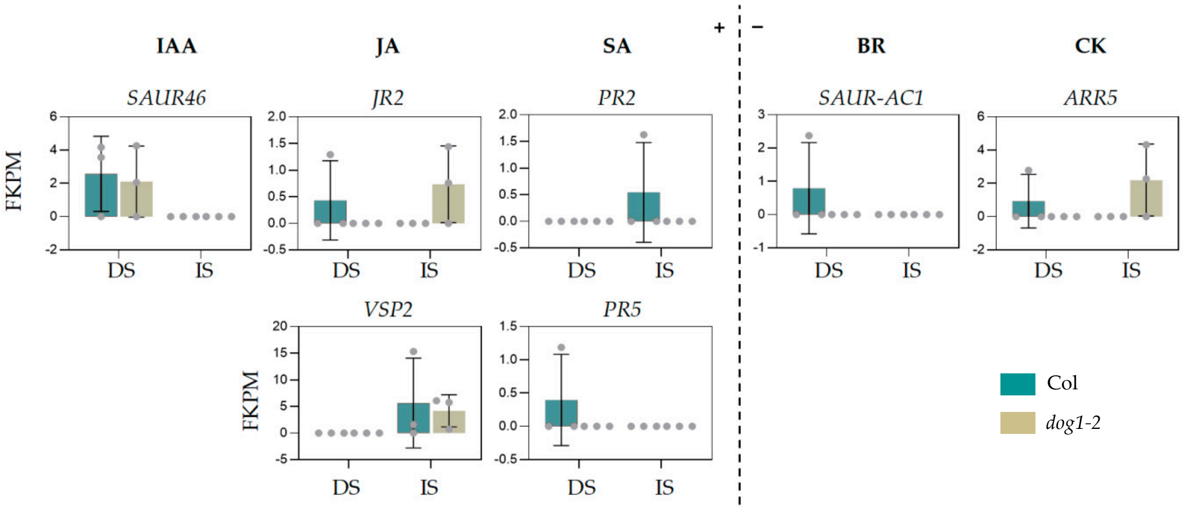

Figure S2. The expression value of marker genes that are not suitable as indicators of hormonal levels within seeds. DS, dry seeds; IS, imbibed seeds. The dashed line divides the phytohormones into two classes: "+" denotes the phytohormones that induce seed dormancy or inhibit seed germination; "-" denotes the phytohormones that release seed dormancy or promote seed germination.

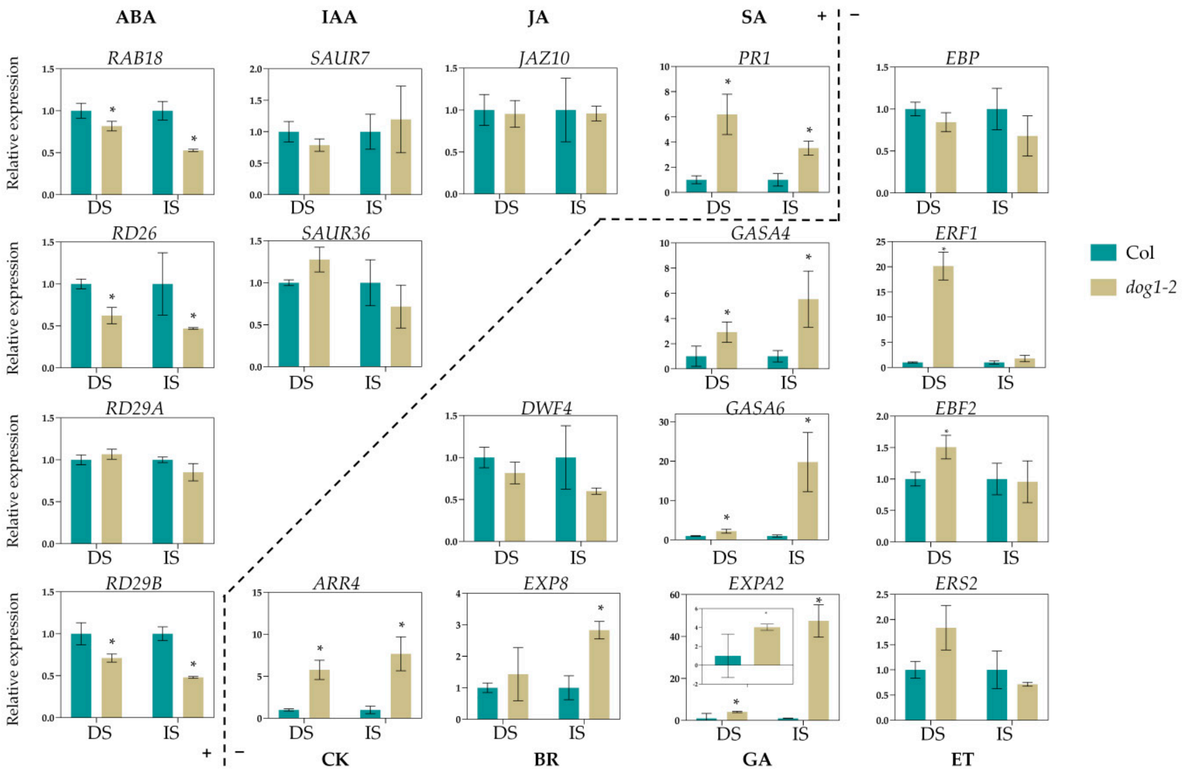

Figure S3: Validation of the Expression of marker genes by RT-qPCR Assays. Relative expression of representative marker genes of the indicated phytohormone pathway in Col and *dog1-2* seeds. DS, dry seeds; IS, imbibed seeds. The dashed line divides the phytohormones into two classes: "+" denotes the phytohormones that induce seed dormancy or inhibit seed germination; " - " denotes the phytohormones that release seed dormancy or promote seed germination. Data represent the average of three replications . *, P<0.05. RT-qPCR primers for each gene is list in Table S3.
